# Acoustic characteristics of sound produced by males of *Bactrocera oleae* change in the presence of conspecifics

**DOI:** 10.1038/s41598-022-16888-8

**Published:** 2022-07-29

**Authors:** Anastasia Terzidou, Nikos Kouloussis, George Papanikolaou, Dimitrios Koveos

**Affiliations:** 1 Laboratory of Applied Zoology and Parasitology, School of Agriculture, Aristotle University of Thessaloniki, 541 24 Thessaloniki, Greece; 2 Laboratory of Electroacoustics and Television Systems, Department of Electrical and Computer Engineering, Aristotle University of Thessaloniki, 541 24 Thessaloniki, Greece

**Keywords:** Zoology, Ecology, Agroecology, Behavioural ecology

## Abstract

Males of the olive fruit fly *Bactrocera oleae* vibrate and stridulate their wings at dusk producing sounds different from flight sounds with no confirmed behavior role. We recorded and performed a temporal-spectral analysis of this sound. Sound produced by male wing vibration/stridulation consists of intermittent pulses of highly variable duration and of fundamental frequency of around 350 Hz. Flight sound has a much lower fundamental frequency of approximately 180 Hz. Males begin to display wing vibration and sound production at the beginning of their sexual maturity at the 5th day of their age. This behavior is more pronounced in the presence of another conspecific male and observed less in male–female pairs or in solitary males. Broadcasts of the recorded sound did not attract flies of either sex. The highest fundamental frequency was found in association with wing vibrations emitted by male–male pairs, followed by those emitted by male–female pairs and then solitary males, which showed the lowest frequency values. The mean pulse duration and interpulse interval were shorter in male–male pairs than in male–female pairs. We assume that the male wing vibration and the produced signal, apart from its possible role in the courtship of the females, could also be associated with male–male interactions for territorial and rival activities, for which further experiments are required.

## Introduction

Male wing vibration is a behavior linked to courtship in many Tephritid flies^1^ like *Bactrocera cucurbitae*^2^, *Bactrocera tryoni*^3^, *Anastrepha suspensa*^4^ and *Ceratitis capitata*^5^ that spread male pheromone with this behavior. Wing sexual dimorphism in Tephritidae -where the male wing possesses microtrichia along the Cu + A1 vein and is also wider than the female wing- is considered an adaptation to serve this purpose^6,7^.

For *Bactrocera oleae*, the sound produced by wing vibration/stridulation of microtrichia against the abdominal pecten is audible to the human ear and has been described and recorded^8–10^. However, the role of this produced sound on mating and the reproductive success of *B. oleae*, if any, has not yet been clarified. The courtship sounds of *C. capitata*^11^, *B. tryoni*^12^, *B. cucurbitae*^13^ and *A. suspensa*^14^ have been recorded and analyzed acoustically. Broadcasts of recorded calling sounds of *C. capitata* males elicit increased captures of females in traps^15^. Caribbean fruit fly (*A. suspensa*) females respond to the calling sounds of a male conspecific and their response was enhanced when they had been previously exposed to the male pheromone^16^. Acoustical parameters of wing vibration can differ according to the social context i.e., the presence of a conspecific in *A. suspensa*^17^ and in the parasitoid wasp *Psyttalia concolor*^18^.

In tephritid flies, physical and olfactory cues play an important role during courtship and mating rituals. Especially olfactory stimuli are crucial during the mate-searching phase^1^. The male wing vibration/stridulation is associated with the production of visual, olfactory, and auditory stimuli^19,20^, in *B. dorsalis*^21^, *B. tryoni*^22^, *B. cucurbitae*^23^ and *B. oleae*^1^. The release and perception of semiochemicals are often accompanied by a range of intense behavioral interactions, including wing vibration, buzzing and head rocking^24^.

Physical cues (i.e. semiophysicals) may include substrate-borne vibrations, sounds, lights and colors^25^. Bands and spots on the wings of many tephritid species are visual cues that, particularly when sexually dimorphic, could play a role in courtship and mating sequences. Also, other body parts that are brightly colored and/or patterned could have communicative functions in sexual behaviour^1^. In mating communication, vibrational signals allow the expression of many behavioral traits that also carry information of individual fitness. A species can produce a variety of signals, which are characterized by certain spectral and temporal features that eventually drive mate choice. Signals are also significant in intra-sexual competition (rivalry between males), in inter-species or antagonistic interactions^26^.

The olive fruit fly has been an important pest of olives in Mediterranean countries for at least 2000 years^27^ and in 1998 it was first detected in California^28^. The distribution of the pest now covers the Mediterranean basin, north and sub-Saharan Africa, south-west Asia and North America^29^. Mating in *B. oleae* occurs during the last hours of the photophase and at that time high-pitched sounds have been recorded in cages with males^30^.

It is common in tephritid male flies to fight for territories before the initiation of courtship behavior^31^. Olive fruit fly males form swarms on the windward side of trees in late afternoon and within the swarm, displays of aggressive territoriality take place between males in order to exclude male conspecifics before the beginning of courtship displays. Behaviors like synchronous wing waving, fast running towards the opponent, pouncing and boxing on the head and thorax of the opponent are part of the aggressive actions between males^32^. Tephritid males, when engaging in synchronous wing waving, hold their wings perpendicular to the long axis of their body and move them up and down repeatedly, often while moving from side to side in front of the other male (a behavior that differs greatly from the wing vibration during courtship)^33^. In *B. tryoni*, male synchronous wing waving is a highly significant behavioral difference between successful and unsuccessful males and may represent either a simplified courtship dance used by females to recognize males, or an indication of male activity that improves their mating success^34^.

Clarifying the involvement of male sounds in the courtship ritual, mating and reproductive success could be crucial for the improvement of the mass rearing of the fly and the application of the SIT method^35,36^.

The mechanism of sound perception in Tephritid flies is unknown. In mosquitoes and *Drosophila* species auditory stimuli are received by their antennas, which act as acoustic oscillators^37^. They can detect the particle velocity component of the sound, which attenuates rapidly with increasing distance from the source. Therefore, insects with antennal hearing have evolved to detect low-frequency sound sources in the near field (some tens of centimeters)^38^. In this type of acoustic signal, the temporal components of the sound are important for species recognition and sexual selection^39^. In the olive fruit fly*,* courtship with wing vibrations of higher frequency resulted in successful matings. The parameters of male olive fruit fly wing vibration were acquired via video captures and frame-by-frame analysis^7^.

In the present work, our aim was firstly to obtain a high-quality recording of the sound produced by the male olive fruit fly wing vibration/stridulation and analyze the air-borne component of the vibration. We then tested in broadcasting bioassays the attraction and arrestment effect of the sound on the olive fruit flies. Secondly, we explored how the male’s wing vibration behavior is affected by the presence of conspecifics and we compared the acoustic parameters of sounds produced by solitary males with those produced in the presence of a male or female conspecific.

## Results

### Test 1: Audio recording of male wing vibration/stridulation and flight sounds

**Figure 1:**
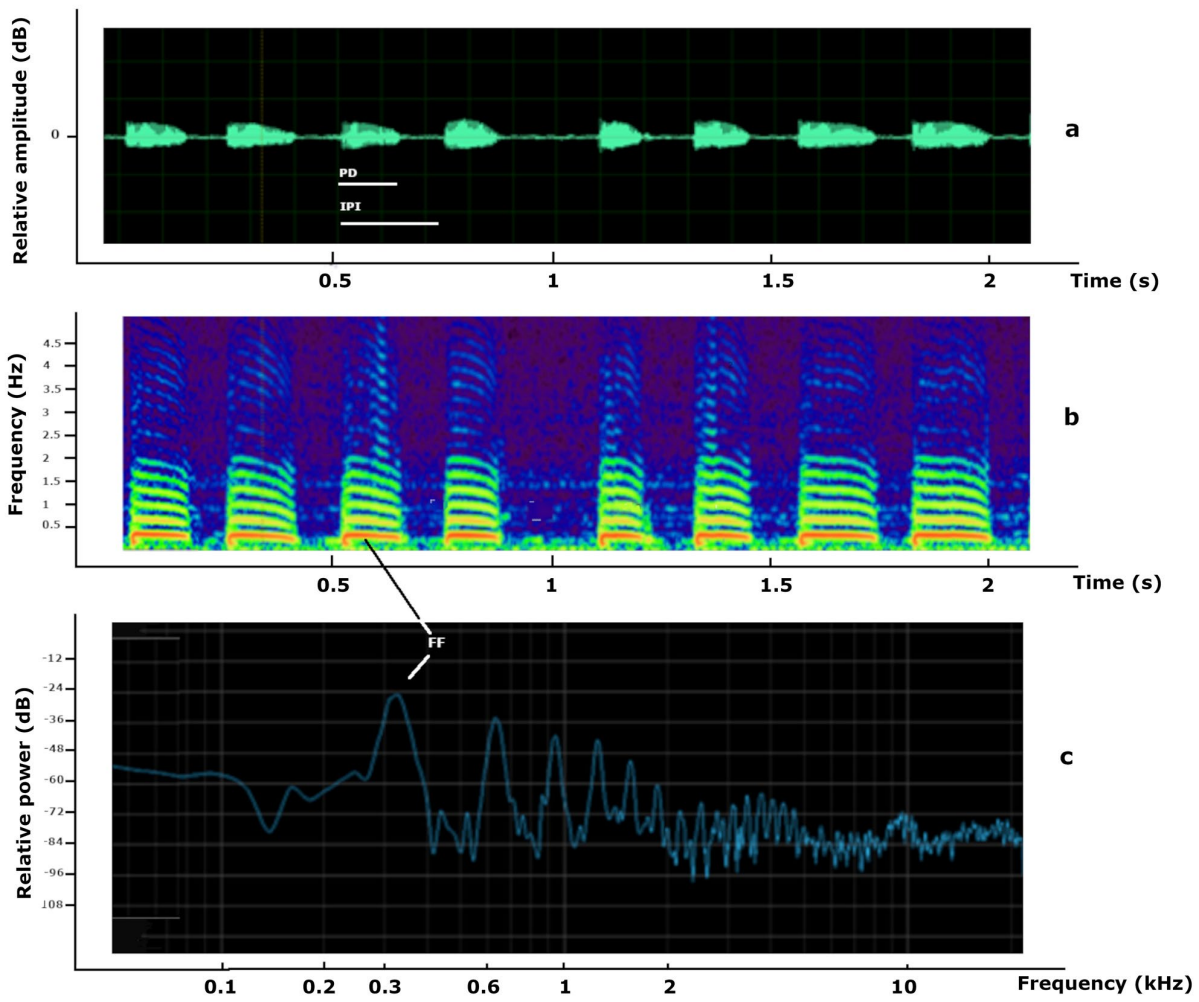
Waveform (**a**), spectrogram (**b**), and frequency analysis (**c**) of the sound produced by the male olive fruit fly vibration/stridulation. In (**a**) the sound waveform of eight pulses is shown of about 0.15 s duration each. Pulse duration (PD) and interpulse interval (IPI) are depicted with bars. The horizontal axis corresponds to time (s) and the vertical to the relative amplitude of the sound in decibels (dB). In (**b**) the spectrogram of the same sound is shown, where the vertical axis corresponds to frequency (in kHz) and the horizontal to time (s). The more intense colored areas of the spectrogram are related to the higher power of the corresponding sound. The maximum power is concentrated at about 380 Hz which is the fundamental frequency (FF) of the sound. In (**c**) the frequency analysis of the sound is shown, where the vertical axis corresponds to the relative power (in dB) and the horizontal axis to the frequency (Hz). The first peak of the spectrum is the fundamental frequency of the sound, at about 380 Hz, followed by harmonics of lower power, at approximately 600 Hz, 900 Hz and 1200 Hz.

The sound produced by the male wing vibration/stridulation consisted of a series of intermittent pulses of a duration ranging from 0.06 to 1.58 s and of a fundamental frequency ranging from 300 to 394 Hz. The fundamental frequency was also the dominant frequency (the one with the maximum power level) and there were higher harmonics of lower power, at about 800 Hz, 1200 Hz, up to 10 kHz. (Fig. 1). While no signals were found in the females’ box, the males’ only box had signaling behavior. The mean fundamental frequency of the flight sound was similar between males (180.94 Hz) and females (179.10 Hz) and they did not differ significantly (*t* = 0.662, *P* = 0.516, n = 30). The waveform, spectrogram and frequency analysis are shown in Supplementary Fig. S3 in Supporting Information.

### Test 2: Response of olive fruit flies to broadcasts of recorded wing vibration

There was no significant statistical difference between the mean number of flies that remained at the area near the sound source (7.75 ± 1.68 females, 9.40 ± 2.77 males) and at the opposite side of the experimental arena (8.85 ± 1.92 females, 7.70 ± 3.01 males) after 1 h of broadcast bioassay for both sexes (*t* = 1.923*, P* = 0.06 for females, *t* = − 1.855*, P* = 0.07 for males, n = 20 for each sex).

### Test 3: Wing sexual dimorphism

Our experiments showed that the mean wing length was significantly longer in females (4.59 ± 0.22 mm) than in males (4.49 ± 0.15 mm) (*t* = 3.233, *P* = 0.003, n = 30). Wing surface (size) was significantly larger in adult females (6.14 ± 0.54 mm^2^) than in males (5.87 ± 0.32 mm^2^) (*t* = 3.241, *P* = 0.003, n = 30). Yet, the wing width did not differ between females (2.24 ± 0.09 mm) and males (2.27 ± 0.12 mm). However, the curvature index of the Cu1 cell was significantly longer in males (143.88 ± 10.21 mm) than in females (104.16 ± 11.81 mm) (*t* = 6.238, *P* < 0.0001, n = 30) (Table 1 and Supplementary Fig. S1 and S2 in Supporting information).

**Table 1:** Anatomical characteristics of male and female wings of *B. oleae.*

Wing	Mean values (SD)			
	Length^a^ (mm)	Width^b^ (mm)	Total area^a^ (mm^2^)	Curvature index^b^ (μm)
Male	4.49 (0.15)	2.24 (0.09)	5.87 (0.32)	143.88 (10.21)
Female	4.59 (0.22)	2.27 (0.12)	6.14 (0.54)	104.16 (11.81)
*p*-value	0.003	0.13	0.003	< 0.0001

^a^For the length and total area of wings, the variances were unequal, and the non-parametric Mann–Whitney test was performed for comparison.

^b^For the width and curvature index, the variances are equal and the Student’s *t*-test (two-tailed, unpaired data) was performed. *n* = 50; Level of significance α = 0.05.

### Test 4: Observation of male wing vibration behavior and mating in relation to age and time of day

**Figure 2:**
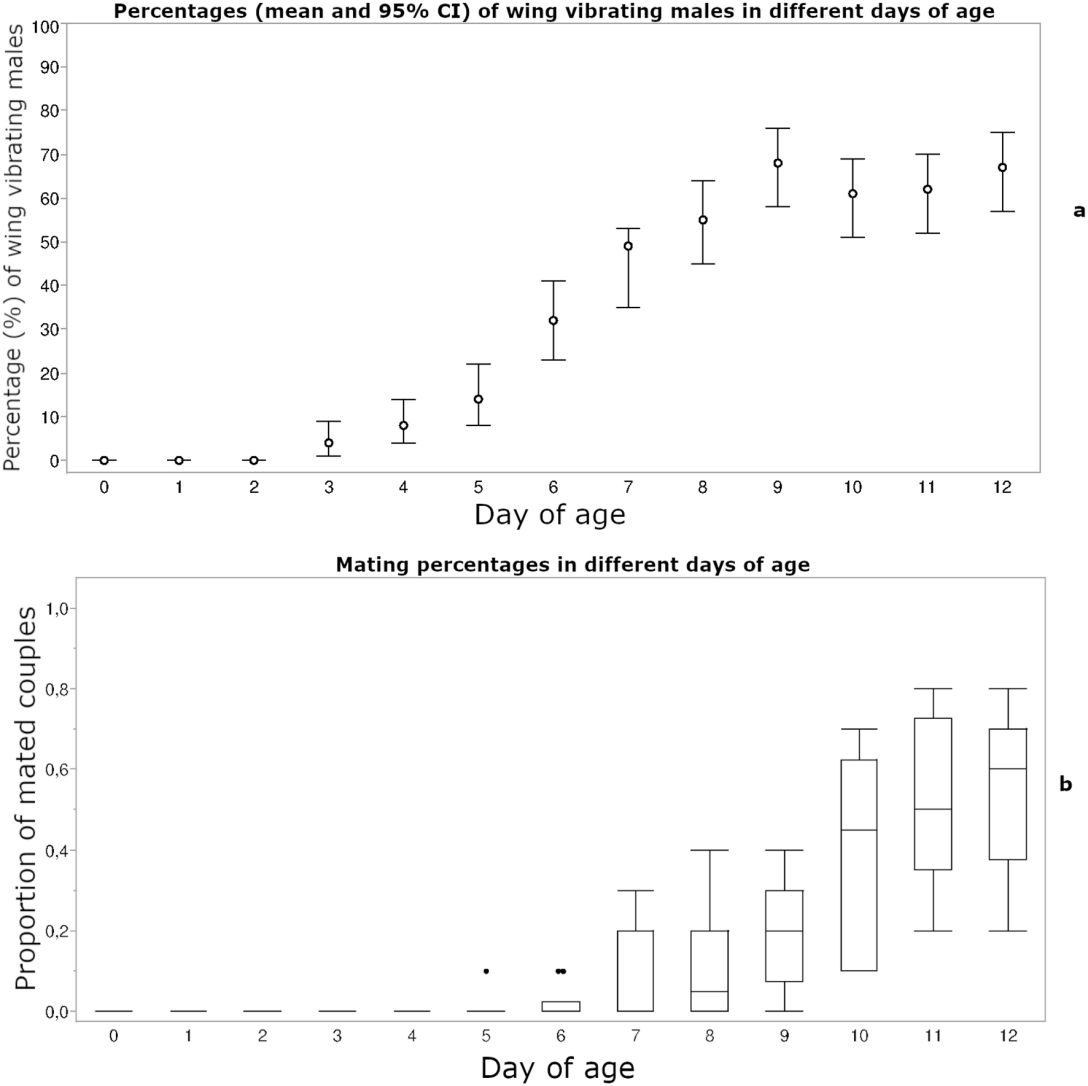
Percentages (mean and 95% CI) of wing vibrating males (**a**) and mating percentages (**b**) in different days of age.

Male olive fruit flies engaged in wing vibration behavior only in the late afternoon hours (18:00–20:00). No wing vibration was observed in the morning or early afternoon hours. The behavior started from the 4th day after adult emergence when 4% (95% CI 1–9%) of males in male–male pairs were engaging in wing vibration. Maximum occurrence of wing vibrating males occurred on the 10th day of age, when 68% (95% CI 58–76%) of males were engaging in this behavior, which continued until the 12th day of age (Fig. 2).

On the 8th day of age, we observed 10% matings and a maximum of 56% matings was reached on the 12th day of age (Fig. 2). The time that each mating started during the day was also noted (Supporting information, Supplementary Fig. S4). First matings occurred 4 h before the end of the photophase and the maximum was reached 2–2.5 h later.

### Test 5: Male wing vibration parameters and behaviors in different social situations

**Figure 3:**
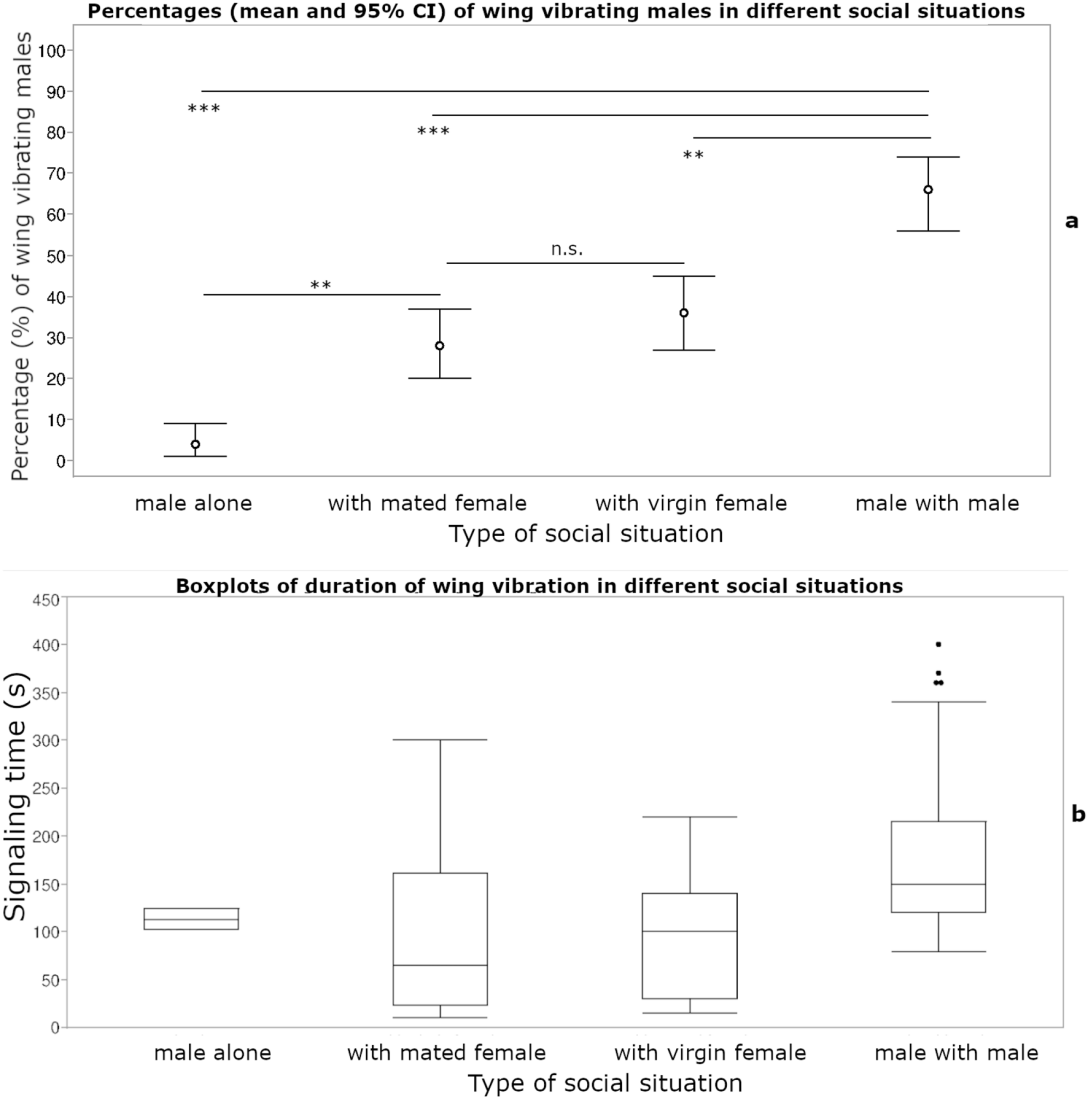
Percentages (mean and 95% CI) of wing vibrating males (**a**) and boxplots of duration of wing vibration (**b**) in different social situations.

In single males, 4% (95% CI: 1–9%) of the individuals were observed engaging in wing vibration during the last 2 h of the photophase and the mean time spent signaling was 113.00 ± 15.55 s. In the presence of a virgin female, 28% (95% CI: 20–37%) of males were observed engaging in wing vibration for a mean time of 94.70 ± 65.79 s. Copulations followed quickly in the majority of observations, but when females were not immediately receptive for mating, the males were seen wing-vibrating in several observations and attempted unsuccessful copulations. In the presence of a mated female, 36% (95% CI: 27–45%) of males were observed wing vibrating for a mean time of 100.55 ± 97.60 s and no copulations occurred. In male–male pairs, 66% (95% CI 56–74%) of males engaged in wing vibration for a mean time of 145.73 ± 72.13 s (Fig. 3).

Rivalry behaviors like fighting (including boxing with front legs, often followed by dropping to the bottom of the cage) were observed in 70% (95% CI: 60–78%) of the male–male pairs. 48% (95% CI: 38–57%) of the males attempted copulation with the other males. 30% (95% CI: 21–39%) of the males were observed doing the synchronous wing waving behavior, while they were at opposite positions and with eye contact with each other (Table 2). In single males, the majority were stationary during the observation period, engaging in feeding or cleaning activities and only an extremely low proportion of 4% (95% CI: 1–9%) were seen wing vibrating at 1–2 occasions.

**Table 2:** Proportion (mean and CI) of males exhibiting antagonistic behaviors in male–male pairs.

Type of behavior in male–male pairs	Mean proportion (95% CI)
Synchronous wing waving	0.30 (0.21–0.39)
Wing vibration	0.66 (0.56–0.74)
Fighting	0.70 (0.60–0.78)
Attempted copulation	0.48 (0.38–0.57)

Fighting: % of pairs engaging in this behavior during the observation period.

Synchronous wing waving, wing vibration, attempted copulation: % of individual males engaging in the behavior.

The mean fundamental frequency (FF) varied depending on the social context of the male i.e., mean (± SD) fundamental frequency was low in single males (324.40 ± 17.10 Hz), increased when males were maintained in pairs with mated or virgin females (352.93 ± 11.43 Hz and 347.68 ± 19.27 Hz respectively) and was very high when males were maintained with other males (365.23 ± 15.78 Hz). One-way Anova provided evidence of significant differences in fundamental frequency according to the social context (F = 11.58, *df* = 3, *P* > 0.0001). Tukey post-hoc test showed that the FF of wing vibration in male–male pairs was higher than in male-virgin female pairs and solitary males (Table 3).

**Table 3:** Fundamental frequency (FF), pulse duration (PD), interpulse interval (IPI) (mean (SD), median, range and sample size) of the sound produced by the wing vibration/stridulation of a single virgin male and in the presence of a mated female, a virgin female and a male.

Variable	Social context	Mean (SD)	Median	Min–Max	*n*
**FF (Hz)**	single ♂	324.40 (17.10)c	317	307–353	10
	♂ + mated ♀	352.93 (11.43)ab	352	335–369	10
	♂ + virgin ♀	347.68 (19.27)b	349	300–373	16
	♂ + ♂	365.23 (15.78)a	363	344–394	13
*p-value*		< 0.0001			
**PD (s)**	single ♂	0.15 (0.06)ab	0.16	0.062–0.28	10
	♂ + mated ♀	0.33 (0.38)a	0.22	0.11–1.38	10
	♂ + virgin ♀	0.28 (0.36)a	0.16	0.07–1.58	16
	♂ + ♂	0.13 (0.07)b	0.11	0.066–0.32	13
*p*-value		0.0462			
**IPI (s)**	single ♂	0.33 (0.06)ab	0.33	0.23–0.43	10
	♂ + mated ♀	0.49 (0.37)a	0.37	0.30–1.54	10
	♂ + virgin ♀	0.49 (0.46)a	0.36	0.24–2.17	16
	♂ + ♂	0.27 (0.07)b	0.27	0.27	13
*p*-value		0.0078			

Means within the same group followed by different letters are statistically different (level of significance α = 0.05).

For FF: One-way Anova and Tukey post-hoc test.

For PD and IPI: Kruskal–Wallis H test followed by Mann–Whitney U test pairwise tests.

The mean (± SD) pulse duration (PD) varied from 0.15 ± 0.06 s for single males, 0.33 ± 0.38 s for males with mated females, 0.28 ± 0.36 s for males with virgin females and 0.13 ± 0.07 s for male–male pairs. Kruskal–Wallis test showed a slightly significant difference between treatments (*H* = 7.990, *df* = 3, *P* = 0.0462). Mann–Whitney post-hoc test for pairwise comparisons showed that PD in male–male pairs was shorter than in male–female pairs (mated or virgin) (Table 3).

The mean (± SD) interpulse interval (IPI) was very low (0.27 ± 0.07 s) in the presence of another male and increased in the presence of a mated (0.49 ± 0.46 s) or virgin female (0.49 ± 0.46 s) or solitary males (0.33 ± 0.06 s). Kruskal–Wallis test showed that there was a significant difference between treatments (*H* = 11.879, *df* = 3, *P* = 0.0078) and Mann–Whitney post-hoc test for pairwise comparisons showed that IPI was of shorter duration in male–male pairs than in male–female pairs (mated or virgin) (Table 3).

## Discussion

In our work, we present a for the first time a high-quality digital audio recording of this male-produced sound of *B. oleae* and describe its temporal and spectral characteristics. The sound produced by wing vibration/stridulation is different than that of the flight wingbeat and consists of trains of pulses of variable duration and fundamental frequency (FF) that can vary from 300 to 390 Hz, as it was observed for *B. cucurbitae*^40^. In addition, higher harmonics are also present at multiples of the FF, as it was reported by Feron and Andrieu^9^ and Rolli^10^. Interestingly, male flies were engaging in wing vibration/stridulation even without the presence of female flies in their proximity.

Sexual communication in Tephritidae is multimodal and the auditory stimuli alone did not prove enough to elicit a behavioral response. In our experiments no phonotaxis to the broadcasts was observed. However, it has recently been recognized that communication by substrate-borne vibrations is the most widespread channel of communication in arthropods^25^. In parasitic Hymenoptera, wing fanning performed by males may act as a vibrational stimulus to quieten the female and triggering sexual receptivity^41^. Pea leafminers (*Liriomyza huidobrensis*) produce vibrational signals that convey efficient information to the opposite sex and result in pair formations on substrates, like plant leaves^42^. Also, recent studies on *Drosophila melanogaster* and its two sibling species, revealed that males quiver their abdomens and generate substrate-borne signals to induce female immobility and to enhance the receptivity of females to copulation^43^. For the olive fruit fly, it is possible that during courtship displays on leaves, where attraction could involve visual, olfactory and acoustic cues, vibrations are transmitted through plant surfaces and detected by the legs^44^. It would therefore be interesting to assess whether the signal of male wing vibration that we tested can be attractive to male and female olive fruit flies by using techniques of Biotremology^45^.

The temporal characteristics of produced sound vary depending on the presence of conspecific flies and the pulse duration was from 0.10 to 1.58 s. According to Miyatake and Kanmiya^13^ longer pulse durations are related to male pheromone spraying after its release on the body surface. Similarly, in the braconid parasitoid wasp *P. concolor*, pulse durations were longer in the presence of conspecific females^18^. The variation in the male pulse duration of *B. oleae* in the presence of conspecific individuals could be related to the increased effort exerted by the male when it is near a potential partner.

The sound produced by the wing vibration/stridulation of males may be related to the unique anatomy of their wings compared to females. Earlier studies by Benelli et al^7^ have shown that the total wing size is larger in males than females. By contrast, our results show that the total size of the wings is larger in females than in males, and perhaps this is a regional peculiarity. However, the posterior part of the male wings is larger than the respective part in female wings which may facilitate sound production. This posterior part of the wings is actively involved in sound production by stridulation of microtrichia present on the A1 + CuA2 vein on the pecten of the abdominal tergite (see Supplementary Fig. S1 in Supporting information). Females lack wing microtrichia and abdominal pecten and do not produce any sounds by wing vibration. On the other hand, females are heavier than males and their larger wings may facilitate their flying.

Females of *B. oleae* have immature oocytes in their ovaries in the first 3 to 5 days of their adult life and during this period do not mate. Mating occurs after this period and reaches a maximum on the 10th day of their age^46^. Virgin females produce a multi-component sex pheromone containing four constituents with a synergistic action: 1,7-dioxaspiro[5.5]undecane (DSU) and methyl dodecanoate, a-pinene and nonanal. However, among these compounds, DSU is reported as the most abundant component and exhibits the highest biological activity towards males. Interestingly, young *B. oleae* males also produce DSU in their rectal glands and production of DSU starts from the 1st day after adult emergence, reaches a maximum when gonad maturation is complete, and then decreases to zero by the 11th day. When olive fruit fly males become sexually mature, they start to produce (Z)-9-tricosene, a compound unique to males, which is able to selectively attract females during the close-range phase only^1,47,48^. Here, we show that males begin to perform wing vibration after the first 5 days of their adult life which corresponds with females becoming receptive to mating. Therefore, we consider that the sound produced by male wing vibration is linked to mating. This hypothesis is supported by the fact that wing vibration and mating occur only in the last 4 h of the photophase^30^. In addition, in the presence of females (either virgin or mated), the percentage of males exhibiting wing vibration increases to about 30%. This increase in the percentage of males showing wing vibration in the presence of females was also indicated by Benelli et al^7^ and supports our hypothesis concerning the role of wing vibration in mating.

We found that the fundamental frequency of the pulses varies and is higher in the presence of another male and lower in the absence of another conspecific of either sex. Yet, a higher proportion of males performed the wing vibration when they were together with another male than alone in the cage. This could be due, among other reasons, to an intragroup stimulation to perform the same behavior, as was demonstrated in *C. capitata*^49^. In *B. cucurbitae*^50^ 83% of the males observed in the field were seen wing vibrating when other calling males were in proximity. Males exert more effort for wing vibration and stridulation when they are in groups and this increased wingbeat results in higher fundamental frequencies of the produced sound. In addition, we should not exclude the possibility that this particular sound production, with specific pulse characteristics in the presence of another male, may be related to a territoriality behavior between males. In *B. curcubitae,* when a calling male encounters another male that also engages in wing vibration, each male continues to vibrate the wings before engaging in additional actions (blows with mid and fore legs, head thrusts, attempting mounts)^50^. For some Bactrocera species, Keiser et al.^51^ speculated that males may display wing vibration as a territorial behavior during mating. In olive fruit flies, aggressions between males could serve to maintain territories in which each male fly can perform courtship displays. Aggressive behaviors have been also observed and described between olive fruit fly females on oviposition sites^52^. On the other hand, when in solitary conditions, males demonstrate their courtship behavior with reduced effort, perhaps as a form of training, since in the absence of a conspecific in the proximity, there is a lack of stimuli to provoke this type of behavior. This is in accordance with our results that wing vibration occurs in higher numbers and the fundamental frequency of the produced sound is higher in males maintained in male–male pairs than in single ones.

In conclusion, our research has shown that the sound produced by male wing vibration/stridulation of microtrichia in the abdominal pecten has specific quality characteristics and is quite different than the flight sound. Stridulations and wing vibrations have different spectrogram patterns. The spectrogram in Fig. 1 suggests that most of the acoustic energy in the *B. oleae* signal is concentrated around the fundamental frequency of the wing vibration (300 Hz) and the multiples of the wingbeat harmonics (600 Hz, 900 Hz, 1200 Hz) and decreases rapidly away from the harmonic frequencies. Stridulations, in contrast, have most energy at the harmonics of tooth impact but the energy is fairly uniformly distributed in between (see e.g., the spectrograms of calls from two *Neoscapteriscus* mole cricket species in Rohde et al^53^). The difference in energy output suggests that the microtrichia and abdominal pecten probably do not contribute significantly to the acoustic signal.

Further experiments are now running to consider the ecological significance of this behavior, which, as in other tephritid flies, may be mainly related to the involvement of the lekking system in mating^54–56^. However, the role of the presence of conspecific males and their positive effect on sound production remains unknown. Future research in Tephritidae should focus on the communication via substrate vibrations, as it is recently becoming clear that flies make use of substrate-borne vibrational signals^43,44^.

## Materials and methods

### Insect rearing

Our laboratory colony was established in autumn from field-infested olive fruit collected around Thessaloniki, in northern Greece. Colony flies were kept in wooden cages (30 × 30 × 30 cm) with three sides of metal net and the front side of glass. Insects used in the experiments were reared in the laboratory in olive fruit for no more than three generations. A liquid diet of yeast hydrolysate (MP Biomedicals®), white granulated sugar and tap water (1:4:5 ratio) was provided daily to the stock adult flies.

For all the experiments where virgin flies were used, adult female and male flies soon after their emergence were transferred and maintained separately in plexiglass cages (20 × 20 × 20 cm, 40 flies per cage). In the experiment concerning the male flies’ behavior in the presence of mated females, we used female flies from the stock colony, that were presumed mated, as they had been kept in mixed sex groups since their emergence.

### General observations

Experiments were carried out in laboratory rooms at 25 ± 2 °C and 55 ± 10% relative humidity (RH) and LD 14:10. For the experiments concerning the male wing vibration behavior in relation to age, time of day and the presence of conspecifics, the flies were maintained either individually or in pairs in transparent plastic cups of 400 ml volume as described by Kouloussis et al^57^.

### Test 1: Audio recording of male wing vibration/stridulation and flight sounds

The aim of this test was to obtain a high quality, low-noise recording of the sound produced by the male wing vibration/stridulation to be used for (a) spectral analysis and (b) playback bioassay in test 2, where a possible attraction and arrestment effect on olive fruit flies was investigated. Also, the flight sounds of both sexes were to be compared.

Recording sessions took place in the sound studio of the Laboratory of Electroacoustics, at the Faculty of Electrical Engineering of the Aristotle University of Thessaloniki, Greece. A wooden cage with the experimental flies was placed inside a sound booth with 28 dBA ambient noise level and very low reverberation time (down to 0,4 s). A super gun hyper-cardioid condenser microphone (Seinheiser M67 with K6 powering, frequency range 40–20,000 Hz ± 2.5 dB, Sensitivity 50 mV/Pa ± 2.5 dB) was used for the recording and a precision sound level meter was used for calibration. Both outputs were connected to Digital Audio Workstation with Pro Tools HD environment. Two independent recording tracks were used at 24 bit/48 kHz sampling rate.

A wooden cage (30 × 30 × 30 cm) with metal net sides, containing approximately 70 virgin male and female flies 2–3 days old was placed under the microphone at 10 cm distance from the top side. Both sexes were present in this cage to better imitate natural conditions of courtship during which the male wing vibration is observed. Flies usually rest and perform wing vibration on the topmost side of the cage, due to the proximity to ventilation and light. Recording was continuous for five days, until the male flies started to engage in wing vibration. In addition, we recorded flight sounds of virgin male and female flies maintained separately.

The parameters to be specified were the fundamental frequency and harmonics in the spectrum of the recorded sounds.

### Test 2: Response of olive fruit flies to broadcasts of recorded wing vibration

The aim of this test was to investigate if the broadcasted sound recorded at test 1 had an attraction and arrestment effect on both sexes of the olive fruit fly.

We placed an omnidirectional loudspeaker (M5 Beoplay by Bang and Olufsen, 16.5 cm diameter × 18.5 cm height, Frequency range 37–22,000 Hz, Speaker configuration 1 × 5″ woofer 1 × 1.5″ midrange 3 × 3⁄4″ tweeters, https://www.bang-olufsen.com/en/gr/speakers/beoplay-m5) at one end of a cylindrical net cage placed horizontally (0.5 m diameter × 1.5 m length). At the opposite end of the cylindrical cage a mimic silent control was placed, and the experimental flies were released at the center of the cylinder. The experimental arena was placed parallel to a window in a laboratory room with natural daylight. Experiments took place between 15:00 and 17:00 h in the months of December and January (sun-setting time at approximately 17:15). The loudspeaker broadcasted the recorded sound continuously for 1 h. We chose a high sound intensity level (96–98 dB at 10 cm from the source), because according to Mankin et al^15^, behavioral effects of sound are observed at intensities higher than 93 dB. We released 20 virgin female olive fruit flies (12–16 days old) from an opening at the center of the cylindrical arena and after 1 h we noted the position of the flies in the arena. The same bioassay took place for virgin males of the same age. Each day 2 repetitions were made and we made 20 repetitions for each sex. A total of 400 female and 400 male flies were used.

The experimental arena was divided into three virtual compartments: near the sound source, the middle area and opposite the sound source which serves as control. The number of flies that remained after 1 h of the bioassay in the compartment near the sound source and the opposite compartment were compared with a two-tailed *t*-test (unpaired data).

### Test 3: Wing sexual dimorphism

The aim of this test was to study if there are morphological differences in the wings of male and female flies, that could account for possible differences in flight sounds of the sexes.

The right wings of 50 male and 50 female olive fruit flies were removed with a razor blade and photographed with a camera (Jenoptic Gryphax Naos) connected to a stereoscope (Leica M28). Using the camera software (GRYPHAX version 2.1.0.724), we measured the following parameters: the length, width, total area of the wings and the Cu1 cell curvature index (see Supplementary Fig. S2 in Supporting Information). Wing length and width were estimated using landmarks according to Benelli et al^7^.

### Test 4: Observations of male wing vibration/stridulation behavior and mating percentage in relation to age and time of day

This was a preliminary test aiming to determine from which age and time of the day the male flies begin the wing vibration/stridulation behavior and to associate it with their sexual maturity and courtship rituals which are also related to age and time of the day.

We maintained two virgin male flies in each of 50 individual cages for their first 12 days of age and we observed and scored wing vibration behavior. To observe the behavior of each male fly, they were painted with a different color (non-toxic watercolor was used) on the thorax. Observations were carried out every ten minutes during a 2-h period in the morning, afternoon, and evening at 10:00–12:00, 14:00–16:00 and 18:00–20:00. The daylight period was from 07:00 until 21:00. We measured the proportion of individual male flies that were observed to wing vibrate to the total number of male flies observed at each day of observation.

For the experiments concerning the determination of mating percentages in relation to the age of the flies, ten virgin males and ten virgin females of the same age were maintained in each of 10 plexiglass cages (15 × 15 × 15 cm) and observed from 15:00 until 21:00 for mating. There were ten repetitions for each day of bioassay (from 1st until 12th day of age) and new sets of virgin flies were used every day. When a successful mating occurred, the pair was removed from the cage and the time of the beginning of mating was scored. We determined the proportion of mated couples to the total number of pairs that were maintained in the cages at the beginning of the bioassay.

### Test 5: Male wing vibration parameters and behaviors in different social situations

The aim of this group of experiments was to observe the behavior of virgin males in the presence of another virgin male, virgin female and mated female, focusing on the wing vibration/stridulation frequencies and associated behaviors. We recorded the male wing vibration and observed the behavior of individuals in the presence of a conspecific.

For the recording of the male wing vibration in different social situations, male flies were transferred 1 day after emergence into a laboratory room (temperature 25 ± 2 °C and 55 ± 10% relative humidity (RH) and LD 14:10), where no females were present and kept in plexiglass cages (20 × 20 × 20 cm). On the 8th day of age, they were transferred and maintained inside plastic cups of 400 ml volume (as described in General observations) either individually or in male–male pairs (50 cups for each treatment). In another laboratory room, with the same temperature and photoperiod conditions, virgin male-virgin female pairs and virgin male-mated female pairs were maintained in each plastic cup (50 pairs for each treatment). All virgin flies were 8 days old at the beginning of the experiment whereas mated females were 11–12 days old and had been kept since their emergence in a stock colony cage.

In all treatments, observations were carried out during the last 4 h of the photophase and sounds produced by males vibrating their wings were recorded with the help of a directional condenser microphone (VideoMicMe, Røde, Australia, frequency range 100 Hz–20 kHz, sensitivity 33.0 dB re 1 Volt/Pascal (22.00 mV @ 94 dB SPL) + /− 2 dB @ 1 kHz, https://www.rode.com/microphones/videomicme) connected to a smartphone. When a mating occurred in virgin male-virgin female pairs, individuals were replaced with new ones of the same age and treatment and observations continued the following day. During the observation period, we obtained n = 10 useful recordings of males in solitary conditions, n = 10 in male-mated female pairs, n = 16 in male-virgin female pairs and n = 13 in male–male pairs. Recordings had to be discarded when they were of short duration and of low signal-to-noise ratio.

For the behavioral bioassay, we observed the flies every 10 min during the last 2 h of the photophase. We observed and scored each individual for the following behaviors: (1) wing vibration/stridulation, (2) fighting, i.e. boxing and pouncing, (3) attempted copulation and (4) wing waving according to Benelli^32^. Fighting engaged both individuals, so the parameter measured was the number of pairs observed in a fight. Behaviors of wing vibration, wing waving and attempts of copulation were scored for each individual fly.

In the presence of a female (virgin or mated) the male flies were observed every 10 min for wing vibration and the time spent signaling during the 2-h observation period was scored with a stopwatch. We made the same observations for male flies maintained in the cages without the presence of a conspecific. We observed 50 pairs for each treatment for two consecutive days. Different flies were used each day.

### Calculation of sound temporal and spectral parameters

Recordings were analyzed using the software Praat-doing phonetics by computer v.6.1.39^58^ for defining the pulse duration (PD), inter-pulse interval (IPI) and fundamental frequency (FF). Adobe Audition 3.0 was used for frequency analysis graphs and spectrographs. Fast Fourier transformations (FFT) were calculated on 2048-point time-slices of the waveforms using a Hamming window. In accordance with Joyce et al^59^ the second, middle and second to last pulses were used for measuring PD, IPI and FF and then averaged. Spectral analysis diagrams were computed in Adobe Audition version 3.0 and the figures were made with GIMP 2.10.22.

### Data analysis

Normality and homogeneity were tested with the Shapiro–Wilk test and Levene’s test, respectively. In wing morphology and flight sounds data, the number of samples was n > 30 and comparisons were made with the *t-*test (two-tailed, unpaired data). The acoustic parameters of male wing vibration in the presence of conspecifics were compared with one-way Anova and Tukey post-hoc test when assumptions for normality and homogeneity were met. When they were violated, the non-parametric Kruskal Wallis test was used with the Mann–Whitney post-hoc test for pairwise comparisons.

Proportions were compared with Pearson’s chi-square test. For all tests, the level of significance was α = 0.05. All statistical tests were performed with JMP 14.1.0^60^.

### Consent for publication

All authors read and approved the final manuscript.

## Supplementary Information

Supplementary Information.

## Data Availability

The data that support the findings of this study are available from the corresponding author upon reasonable request.

## References

[1] Benelli G (2014). Sexual communication and related behaviours in Tephritidae: Current knowledge and potential applications for Integrated Pest Management. J. Pest Sci..

[2] Kuba H, Sokei Y (1988). The production of pheromone clouds by spraying in the melon fly, *Dacus cucurbitae* coquillett (Diptera: Tephritidae). J. Ethol..

[3] Fletcher BS (1969). The structure and function of the sex pheromone glands of the male Queensland fruit fly, *Dacus tryoni*.. J. Insect Physiol..

[4] Nation JL (1972). Courtship behavior and evidence for a sex attractant in the male Caribbean fruit fly, *Anastrepha suspensa*. Ann. Entomol. Soc. Am..

[5] Arita LH, Kaneshiro KY (1989). Sexual selection and lek behavior in the Mediterranean fruit fly, *Ceratitis capitata* (Diptera: Tephritidae). Pacific Sci. (EUA).

[6] Briceño, R.D. & Eberhard, W.G. Male wing positions during courtship by Mediterranean fruit flies (*Ceratitis capitata*) (Diptera: Tephritidae). *J. Kansas Entomol. Soc*. 143–47 (2000).

[7] Benelli G (2012). Male wing vibration in the mating behavior of the Olive fruit fly *Bactrocera oleae* (Rossi) (Diptera: Tephritidae). J. Insect Behav..

[8] Feron M (1960). L’appel sonore du mâle dans le comportement sexuel de *Dacus oleae* Gmel [Dipt Trypetidae]. Bull. Soc. Entomol. Fr..

[9] Feron M, Andrieu AJ (1962). Etude des signaux acoustiques du male dans le comportement sexuel de *Dacus Oleae* Gmel (Dipt. Trypetidae). Ann. Epiphyt..

[10] Rolli K (1976). Die akustischen Sexualsignale von *Ceratitis capitata* Wied. Und *Dacus oleae* Gmel. Z. Angew. Entomol..

[11] Webb JC, Calkins CO, Chambers DL, Schwienbacher W, Russ K (1983). Acoustical aspects of behavior of Mediterranean fruit fly, *Ceratitis capitata*: Analysis and identification of courtship sounds. Entomol. Exp. Appl..

[12] Mankin RW, Lemon M, Harmer AMT, Evans CS, Taylor PW (2008). Time pattern and frequency analyses of sounds produced by irradiated and untreated male *Bactrocera tryoni* (Diptera: Tephritidae) during mating behavior. Ann. Entomol. Soc. Am..

[13] Miyatake T, Kanmiya K (2004). Male courtship song in circadian rhythm mutants of *Bactrocera cucurbitae* (Tephritidae: Diptera). J. Insect Physiol..

[14] Sivinski J, Burk T, Webb J (1984). Acoustic courtship signals in the Caribbean fruit fly, *Anastrepha suspensa* (Loew). Anim. Behav..

[15] Mankin RW (2004). Broadcasts of wing-fanning vibrations recorded from calling male *Ceratitis capitata* (Diptera: Tephritidae) increase captures of females in traps. J. Econ. Entomol..

[16] Mankin RW, Petersson E, Epsky ND, Heath RR, Sivinski J (2000). Exposure to male pheromones enhances *Anastrepha suspensa* (Diptera: Tephritidae) female response to male calling song. Fla. Entomol..

[17] Sivinski J, Webb JC (1986). Changes in a Caribbean fruit fly acoustic signal with social situation (Diptera: Tephritidae)1. Ann. Entomol. Soc. Am..

[18] Canale A (2013). The courtship song of fanning males in the fruit fly parasitoid *Psyttalia concolor* (Szépligeti) (Hymenoptera: Braconidae). Bull. Entomol. Res..

[19] Wicker-Thomas C (2007). Pheromonal communication involved in courtship behavior in Diptera. J. Insect. Physiol..

[20] Tan, K.H., Nishida, R., Jang, E.B. & Shelly, T.E. Pheromones, male lures, and trapping of tephritid fruit flies. In: *Trapping and the Detection, Control, and Regulation of Tephritid Fruit Flies: Lures, Area-Wide Programs, And Trade Implications* 15–74 (Springer, 2014).

[21] Poramarcom, R. Sexual communication in the Oriental fruit fly, *Dacus dorsalis* Hendel (Diptera: Tephritidae). Doctoral dissertation. (University of Hawaii at Manoa, 1988).

[22] Ekanayake, D. The mating system and courtship behaviour of the Queensland fruit fly, *Bactrocera tryoni* (Froggatt) (Diptera: Tephritidae). Doctoral dissertation. (Queensland University of Technology, 2017).

[23] Suzuki Y, Koyama J (1981). Courtship behavior of the melon fly*, Dacus cucurbitae* Coquillett (Diptera: Tephritidae). Appl. Entomol. Zool..

[24] Scolari F, Valerio F, Benelli G, Papadopoulos NT, Vaníčková L (2021). Tephritid fruit fly semiochemicals: Current knowledge and future perspectives. Insects.

[25] Nieri R, Anfora G, Mazzoni V, Rossi Stacconi MV (2022). Semiochemicals, semiophysicals and their integration for the development of innovative multi-modal systems for agricultural pests’ monitoring and control. Entomol. Gen..

[26] Cocroft RB, Rodríguez RL (2005). The behavioral ecology of insect vibrational communication. Bioscience.

[27] Daane KM, Johnson MW (2010). Olive fruit fly: Managing an ancient pest in modern times. Annu. Rev. Entomol..

[28] Rice RE, Phillips PA, Stewart-Leslie J, Sibbett GS (2003). Olive fruit fly populations measured in Central and Southern California. Calif. Agric..

[29] Wang X (2021). Exploration for olive fruit fly parasitoids across Africa reveals regional distributions and dominance of closely associated parasitoids. Sci. Rep..

[30] Loher W, Zervas G (1979). The mating rhythm of the olive fruitfly, *Dacus oleae* Gmelin. Z. Angew. Entomol..

[31] Benelli G (2014). Aggression in Tephritidae flies: Where, when, why? Future directions for research in integrated pest management. Insects.

[32] Benelli G (2014). Aggressive behavior and territoriality in the olive fruit fly, *Bactrocera oleae* (Rossi) (Diptera: Tephritidae): Role of residence and time of day. J. Insect. Behav..

[33] Shelly TE (2000). Aggression between wild and laboratory-reared sterile males of the mediterranean fruit fly in a natural habitat (Diptera: Tephritidae). Fla. Entomol..

[34] Ekanayake WM, Clarke AR, Schutze MK (2019). Close-distance courtship of laboratory reared *Bactrocera tryoni* (Diptera: Tephritidae). Austral. Entomol..

[35] Ant T (2012). Control of the olive fruit fly using genetics-enhanced sterile insect technique. BMC Biol..

[36] Estes AM (2012). A basis for the renewal of sterile insect technique for the olive fly, *Bactrocera oleae* (Rossi). J. Appl. Entomol..

[37] Zanini D, Geurten B, Spalthoff C, Göpfert MC, Hedwig B (2014). Sound communication in *Drosophila*. Insect Hearing and Acoustic Communication Animal Signals and Communication.

[38] Windmill JFC, Jackson JC, Pollack G, Mason A, Popper A, Fay R (2016). Mechanical specializations of insect ears. Insect Hearing. Springer Handbook of Auditory Research.

[39] Talyn BC, Dowse HB (2004). The role of courtship song in sexual selection and species recognition by female *Drosophila melanogaster*. Anim. Behav..

[40] Kanmiya K (1988). Acoustic studies on the mechanism of sound production in the mating songs of the melon fly, *Dacus cucurbitae* Coquillett (Diptera: Tephritidae). J. Ethol..

[41] Benelli G, Ricciardi R, Romano D, Cosci F, Stefanini C, Lucchi A (2020). Wing-fanning frequency as a releaser boosting male mating success—High-speed video analysis of courtship behavior in Campoplex capitator, a parasitoid of *Lobesia botrana*. Insect Sci..

[42] Ge J, Wei JN, Zhang DJ, Hu C, Zheng DZ, Kang L (2019). Pea leafminer *Liriomyza huidobrensis* (Diptera: Agromyzidae) uses vibrational duets for efficient sexual communication. Insect Sci..

[43] Mazzoni V, Anfora G, Virant-Doberlet M (2013). Substrate vibrations during courtship in three drosophila species. PLoS ONE.

[44] McKelvey EGZ (2021). Drosophila females receive male substrate-borne signals through specific leg neurons during courtship. Curr. Biol..

[45] Strauß, J., Stritih-Peljhan, N., Nieri, R., Virant-Doberlet, M., & Mazzoni, V. Communication by substrate-borne mechanical waves in insects: From basic to applied biotremology. In: *Advances in Insect Physiology,* vol*.* **61**, 189–307 (Academic Press, 2021).

[46] Mazomenos BE (1984). Effect of age and mating on pheromone production in the female olive fruit fly, *Dacus oleae* (Gmel.). J. Insect Physiol..

[47] Carpita A, Canale A, Raffaelli A, Saba A, Benelli G, Raspi A (2012). (Z)-9-tricosene identified in rectal gland extracts of *Bactrocera oleae* males: First evidence of a male-produced female attractant in in olive fruit fly. Naturwissenschaften.

[48] Canale A, Germinara SG, Carpita A, Benelli G, Bonsignori G, Stefanini C, Raspi A, Rotundo G (2013). Behavioural and electrophysiological responses of the olive fruit fly, *Bactrocera oleae* (Rossi) (Diptera: Tephritidae), to male- and female-borne sex attractants. Chemoecology.

[49] Mcdonald PT (1987). Intragroup stimulation of pheromone release by male mediterranean fruit flies (Diptera: Tephritidae). Ann. Entomol. Soc. Am..

[50] Iwahashi O, Majima T (1986). Lek formation and male–male competition in the melon fly, *Dacus cucurbitae* Coquillett: Diptera: Tephritidae. Appl. Entomol. Zool..

[51] Keiser I, Kobayashi RM, Chambers DL, Schneider EL (1973). Relation of sexual dimorphism in the wings, potential stridulation, and illumination to mating of oriental fruit flies, melon flies, and Mediterranean fruit flies in Hawaii. Ann. Ent. Soc. Am..

[52] Benelli G, Canale A (2016). Aggressive behavior in olive fruit fly females: Oviposition site guarding against parasitic wasps. J. Insect Behav..

[53] Rohde BB (2019). An acoustic trap to survey and capture two neoscapteriscus species. Fla. Entomol..

[54] Shelly TE (2001). Lek size and female visitation in two species of tephritid fruit flies. Anim. Behav..

[55] Niyazi N, Shuker DM, Wood RJ (2008). Male position and calling effort together influence male attractiveness in leks of the medfly, *Ceratitis capitata* (Diptera: Tephritidae): Male attractiveness in leks of *Ceratitis capitata*. Biol. J. Linn. Soc. Lond..

[56] Greenfield MD (2015). Signal interactions and interference in insect choruses: Singing and listening in the social environment. J. Comp. Physiol. A.

[57] Kouloussis NA (2017). Age related assessment of sugar and protein intake of *Ceratitis capitata* in ad libitum conditions and modeling its relation to reproduction. Front. Physiol..

[58] Boersma P, Van Heuven V (2001). Speak and unSpeak with PRAAT. Glot Int..

[59] Joyce AL (2010). Effect of continuous rearing on courtship acoustics of five braconid parasitoids, candidates for augmentative biological control of *Anastrepha* species. Biocontrol.

[60] Sall J (2017). JMP Start Statistics: A Guide to Statistics and Data Analysis Using JMP.

