# Supplementary material for: Acoustic characteristics of sound produced by males of Bactrocera oleae change in the presence of conspecifics
Source: Sci Rep. 2022 Jul 29;12:13086. doi: 10.1038/s41598-022-16888-8 (PMC9338093; doi:10.1038/s41598-022-16888-8)
Supplement: Supplementary file 1 — Supplementary Information. [file 41598_2022_16888_MOESM1_ESM.pdf]

**Acoustic characteristics of sound produced by males of *Bactrocera oleae* change in the presence of conspecifics**

Anastasia Terzidou[a], Nikos Kouloussis[a], George Papanikolaou[b] and Dimitrios Koveos[a,*]

[a] Laboratory of Applied Zoology and Parasitology, School of Agriculture, Aristotle University of Thessaloniki, 541 24 Thessaloniki, Greece

[b] Laboratory of Electroacoustics and Television Systems, Department of Electrical & Computer Engineering, Aristotle University of Thessaloniki, 541 24 Thessaloniki, Greece

Emails: aterzido@agro.auth.gr, nikoul@auth.gr, pap@eng.auth.gr, koveos@agro.auth.gr

*Corresponding author: koveos@agro.auth.gr (Dimitrios Koveos)

## Supplementary material

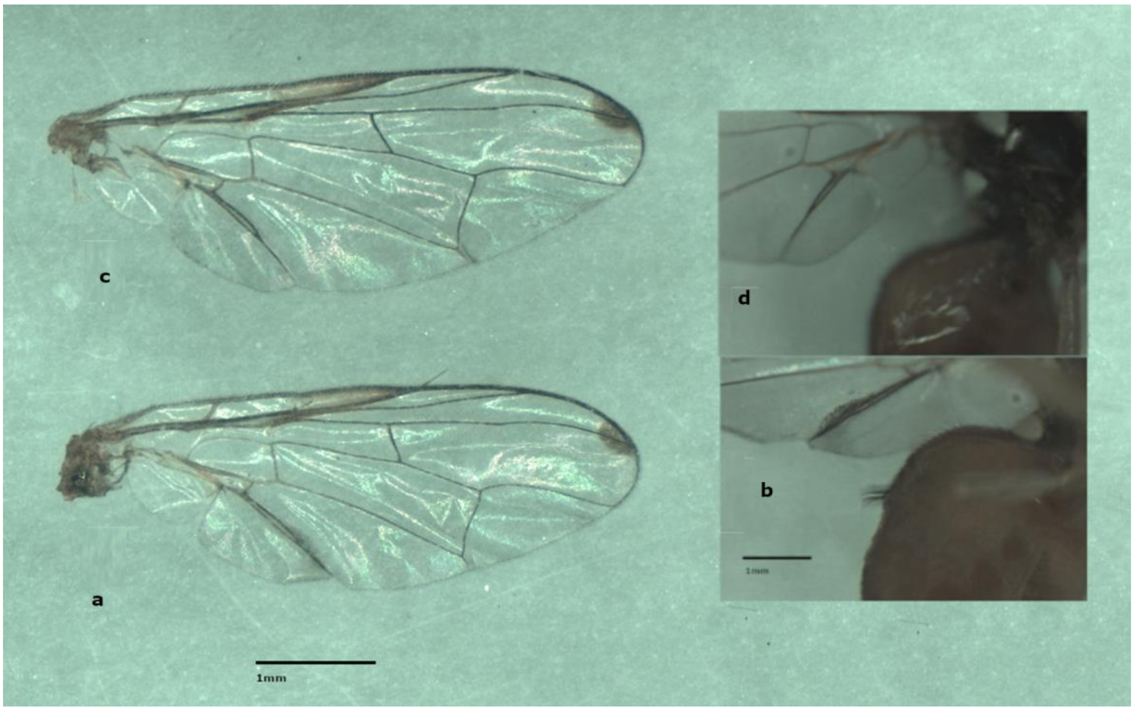


**Supplementary Fig. S1.** Wings and abdominal tergites of females and males of *B. oleae*. Male wings (a) have microtrichia on the A1+CuA2 vein and pecten on the abdominal tergite (b). Females wings (c) lack microtrichia and pecten (d)

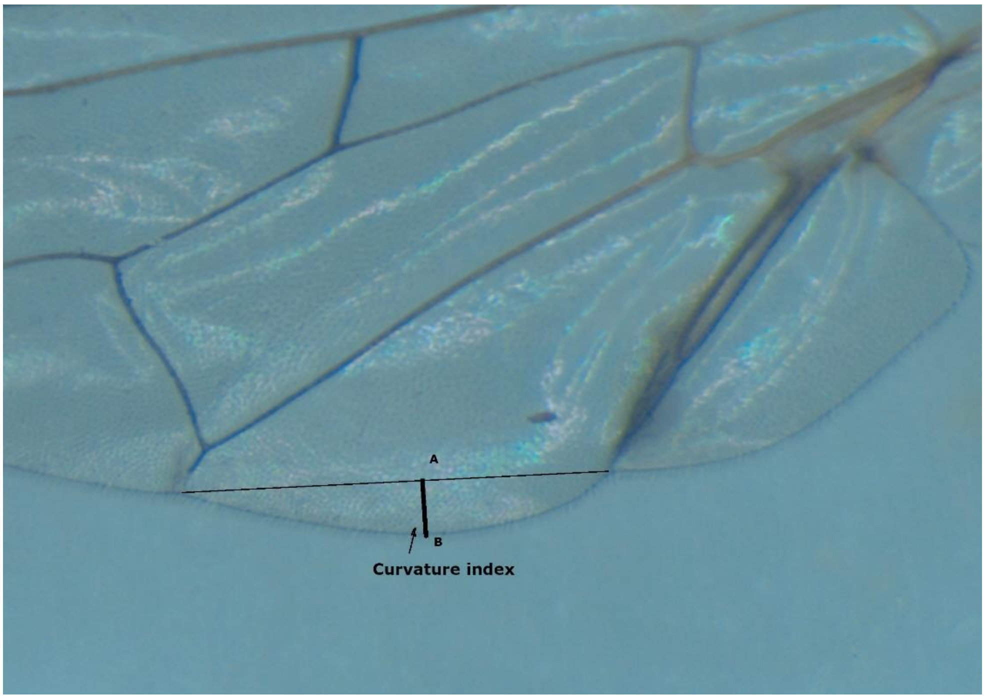


**Supplementary Fig. S2.** The length of the AB line is the curvature index of the Cu1 cell of the olive fruit fly wing.

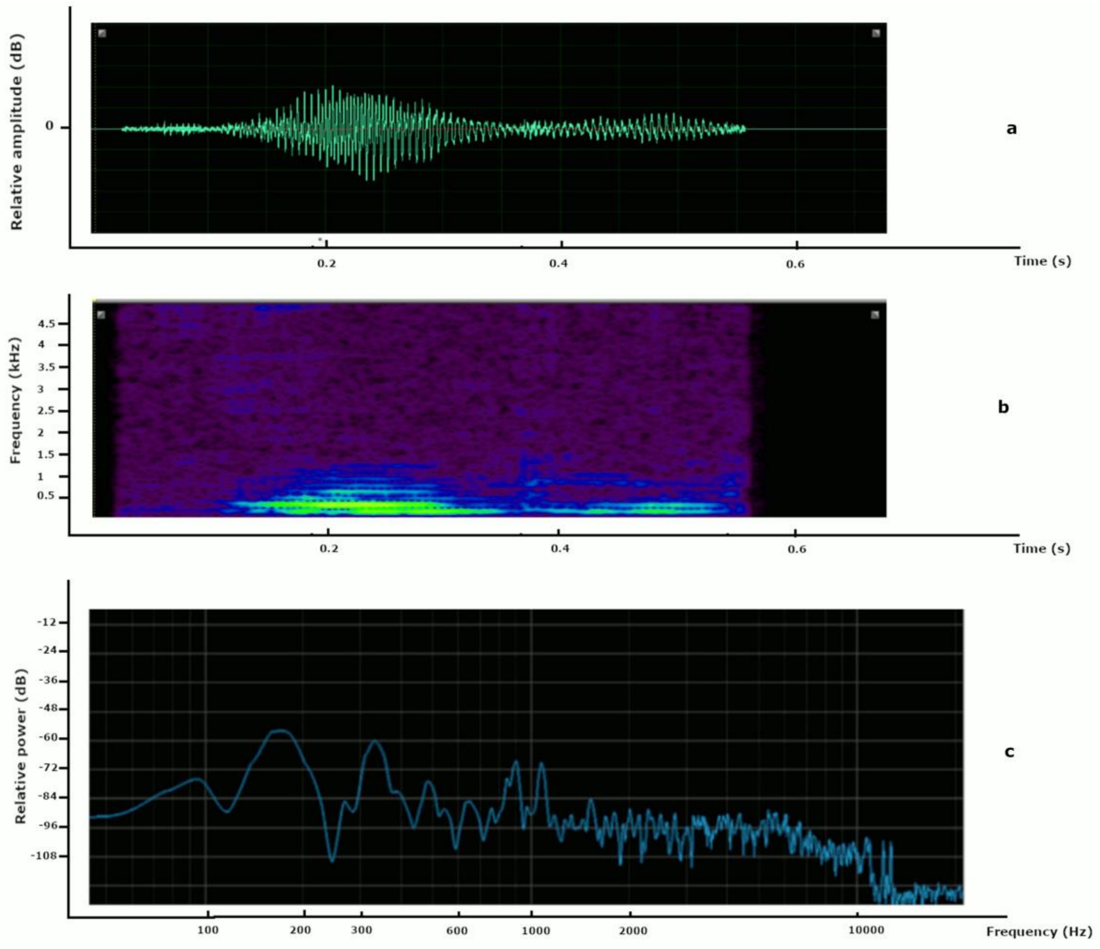


**Supplementary Fig. S3.** Waveform (a), spectrogram (b), and frequency analysis (c) of the sound produced during the flight of the olive fruit fly.

In (a) the horizontal axis corresponds to time (s) and the vertical to the relative frequency of the sound produced during flight take off of an untethered olive fruit fly. The duration of the sound clip is about 0.5 s.

In (b) is shown the spectrogram of the same flight sound. The horizontal axis corresponds to time (s) and the vertical to frequency (kHz). The more intense colored areas of the spectrogram are related to higher power of the corresponding sound. The frequencies around 180 Hz carry the maximum power of the flight sound.

In (c) is shown the frequency analysis of the male olive fruit fly flight sound. The horizontal axis corresponds to frequency (Hz) and the vertical to the relative power of the sound in decibels (dB). The first peak is at about 180 Hz which is the fundamental frequency of the sound and also coincides with the wingbeat of the insect while flying.

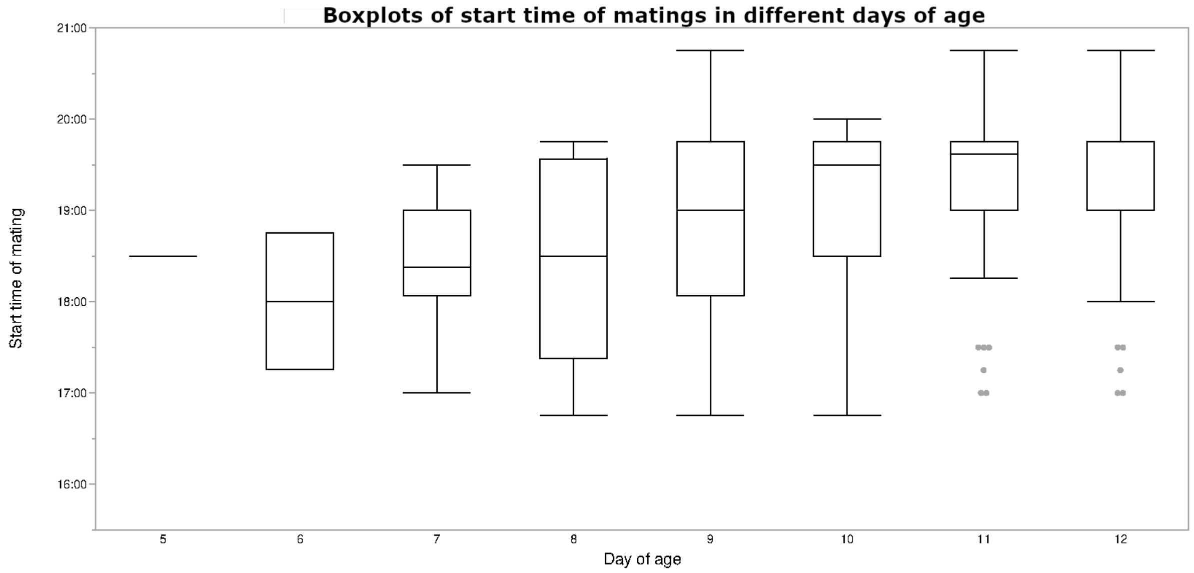


**Supplementary Fig. S4.** Boxplots of start time of matings in different days of age. First matings occurred on the 5$^{th}$ day of age. Bioassay lasted until the 12$^{th}$ day of age). Observations lasted from 15:00 until 21:00 at the end of photophase. Majority of matings started between 18:45 until 19:45.
